# Reevaluating the wave power-salt marsh retreat relationship

**DOI:** 10.1038/s41598-023-30042-y

**Published:** 2023-02-18

**Authors:** L. J. Houttuijn Bloemendaal, D. M. FitzGerald, Z. J. Hughes, A. B. Novak, I. Y. Georgiou

**Affiliations:** 1 Department of Earth and Environment, Boston University, Boston, Massachusetts USA; 2 The Water Institute of the Gulf, New Orleans, Louisiana USA

**Keywords:** Geomorphology, Sedimentology, Physical oceanography

## Abstract

Salt marshes are threatened by rising sea levels and human activities, and a major mechanism of marsh loss is edge retreat or erosion. To understand and predict loss in these valuable ecosystems, studies have related erosion to marsh hydrodynamics and wave characteristics such as wave power. Across global studies, erosion is reported to be largely linearly related to wave power, with this relationship having implications for the resilience of marshes to extreme events such as storms. However, there is significant variability in this relationship across marshes because of marsh heterogeneity and the uniqueness of each physical setting. Here, we investigate the results of individual studies throughout the world that report a linear relationship and add a new dataset from the Great Marsh in Massachusetts (USA). We find that most marsh wave power and erosion data are not normally distributed and when these datasets are properly plotted to account for their distributions, the resulting relationships vary from previously published curves. Our Great Marsh data suggest that events from specific wind directions can have an outsized impact on edge erosion due to their larger fetch and wind speeds. We also find that factors other than wave attack such as edge erosion along tidal channels, can have a measurable impact on retreat rates. We show the importance of maintaining statistical assumptions when performing regressions, as well as emphasize the site-specificity of these relationships. Without calibration of a marsh erosion-wave power relationship using robust regressions for each individual marsh, such a relationship is not fully constrained, resulting in unreliable predictions of future marsh resilience and response to climate change.

## Introduction

Salt marsh edge retreat is a major cause of marsh loss^1^, and when paired with feedbacks between tidal flat erosion and local wind-wave generation, can lead to irreversible marsh collapse even in the absence of relative sea level rise (RSLR)^2^. Marsh edge retreat depends on factors both extrinsic (waves, tidal currents, and tidal flat and channel morphology) and intrinsic (vegetation and soil geotechnical properties)^3–5^. Several empirical and theoretical studies have related marsh retreat to these parameters and determined relationships between wave power or wave height and erosion, allowing for the potential prediction of marsh loss with changing wind/wave climates and RSLR. Marsh edge erosion has been related to both wave thrust and power^6,7^, but here we focus on the latter, as it has been shown to correlate better with erosion rates^8^. We provide a brief assessment of the salt marsh studies that have investigated the relationship between wave power and erosion, however, for a deeper review see Bendoni et al.^9^.

In Rehoboth Bay, Delaware (USA), Schwimmer found an empirical power relationship between wave power (kW/m) and long-term erosion rates (m/yr) and suggested a model for erosion in which increased RSLR outpaces tidal flat and lagoonal sedimentation^10^. Eventually, this process causes an increase in water depth and, thus, larger wave heights and celerities, resulting in increased wave power and ensuing erosion^11^.

Using dimensional analysis, Marani et al. derived a theoretical linear relationship between volumetric retreat rate (lateral shoreline retreat * the height of the marsh bank, in m^2^/yr) and mean incident wave power (wave power projected along the orthogonal direction of the marsh face, in W/m)^12^. The theoretical linear relationship was supported with data from Venice Lagoon (IT); the proportionality constant linearly linking volumetric retreat and incident wave power was shown to be site-specific due to intrinsic marsh properties. Sanford and Gao^13^ investigated spatial correlations between wave power and retreat in the Maryland Chesapeake Bay and found the relationship suggested by Marani et al.^12^ fit reasonably for their data as well. Moreover, Sanford and Gao^13^ suggested several modifications to the Marani et al.^12^ relationship by including 1. dry bulk density; 2. a critical wave power threshold for erosion, though it may be less applicable to marshes than to shoreline banks such as cliffs; and 3. a wave-averaged time-dependent water depth instead of using sea level at mean tide.

Using retreat rates derived from aerial imagery spanning 50 years, McLoughlin et al. confirmed a linear relationship between long-term volumetric erosion rates and wave power in the Virginia Coast Reserve (VCR; USA)^14^. However, the relationship was not significant for individual segments of the marsh shoreline because of considerable within-site variability in erosion, likely due to intrinsic factors affecting erodibility (see^15^). Another study focusing on the VCR also found a linear relationship between retreat rates and wave power, using retreat rates derived from shoreline GPS surveys spanning 3 years, as well as from aerial imagery from 2002 and 2009^8^. However, they found a weak to no correlation between spatial variations in erosion rates and the spatial distribution of wave energy and attributed the variability to local marsh resistance and mass failure processes.

In Venice Lagoon, Bendoni et al. looked at the relationship between volumetric retreat (m^2^/yr) and wave power (W/m) including and excluding mass failures using monthly erosion data covering 1.5 years^16^. They found a linear correlation between short-term retreat and wave power only when excluding mass failures. More recently, Tommasini et al. investigated the temporal geomorphic evolution of the Venice Lagoon over centuries and the evolution of related wind-wave fields responsible for erosion, showing positive feedback between morphological modifications and changes in the wind-wave fields^17^. Their results further emphasized a strong linear relationship between volumetric marsh retreat and incident mean wave power. The linear relationship for Venice Lagoon was confirmed, not just at yearly and centuries-long time scales, but also on monthly time scales as well as at the scale of single storm surge, though the strength of the relationship varied with temporal scale and inclusion of mass failures due to intrinsic factors^18^.

Leonardi et al. synthesized wave power and marsh retreat data from around the world and determined that the data follow a unique and universal linear relationship^6^:

$${E}^{*}= {a}^{*}{P}^{*}, {a}^{*}=0.67,$$

where E* is the dimensionless erosion rate, calculated as the field measurements of retreat rate divided by the average retreat rate for the specific marsh, P* is the dimensionless wave power, calculated as the field measurements for wave power divided by the average wave power for the specific marsh, and a* is a constant that incorporates intrinsic marsh properties. Following this relationship, Leonardi et al. argued that erosion is a continuous process that occurs even under low wave energy conditions, and that due to the linear nature of the relationship, strong storms do not result in catastrophic collapse^6^.

However, studies of marshes have continually emphasized the site-specific variations in the retreat relationships, highlighting potential limitations of a broader application of a generalized relationship. To obtain comparable relationships between erosion and wave power, Bendoni et al. re-analyzed the results from these studies to make them dimensionally consistent (m^2^/yr for erosion and W/m for wave power), illustrating the differences in proportionality coefficients that link these linear relationships^9^. The range of variability in these coefficients suggests that local site characteristics heavily influence the relationship, as well as differences in how wave power was calculated.

Additionally, erosion and wave power at each marsh exhibit different probability density distributions with different magnitudes; for example, as the average erosion rate increases, the frequency-magnitude distribution may have a more normal distribution, while sites with lower average erosion rates can exhibit a long tail of erosion events that result in a more log-normal distribution^8^. These different distributions require different statistical treatment, which were not always applied in previous studies, and which can alter the resulting regressions and derived relationships.

In this study, we reevaluate the universal linear relationship between wave power and marsh retreat. Employing a novel, local dataset from the Great Marsh, Massachusetts (USA), we assess the theory of a global relationship and find non-normally distributed erosion and wave power data and a site-specific, nonlinear relationship between these parameters. When expanding the analysis to previous datasets, building off the global dataset used by Leonardi et al.^6^, the linear relationship fails to describe all datasets, individually or in aggregate. As a result, we reevaluate the conclusion that marshes are inherently resilient to storms due to a linear wave power-retreat relationship; in some cases, storms, in addition to frequent, moderate events, dominate marsh edge erosion, and the nonlinearity of the relationship between wave attack and erosion suggests a vulnerability to high energy events. We emphasize the site-specificity of these relationships, and that parameters used to best explain retreat in one area might not be applicable to another. We further highlight the importance of treating the data in a statistically appropriate manner and transforming the data, if needed, before deriving models, to allow for robust statistics and conclusions.

## Results and discussion

### Relationship between global retreat rates and wave power

Though broadly covered under the umbrella term of “wave power,” the wave parameters used in studies are often subtly different from each other, which can affect the magnitude of wave power presented. For example, Schwimmer calculated wave power for a range of wind speeds and directions and then adjusted it by wind frequency for each speed and direction used^10^, whereas Marani et al. calculated hourly incident wave power and averaged it over a year-long tidal record^12^. McLoughlin et al. provides a more detailed analysis on the impacts of different approaches to calculating wave power, showing that calculations could differ by a factor of 4^14^. Thus, when comparing different datasets, normalization is important to reduce between-marsh differences in the way the parameters were calculated and affected by specific marsh attributes. Following the normalization approach used in Leonardi et al.^6^, dimensionless erosion (E*) and wave power (P*) are used for the global analysis (see Methods).

It is important to note that the meanings of dimensionless wave power and erosion are different than the non-normalized parameters. The E* values are the normalized erosion values, meaning that about half of the observations are greater than 1 and half are less than 1; each individual erosion value is a measured erosion rate at a specific location in the marsh based on field data. The P* values are the normalized wave power values, also meaning that about half of the observations are greater than 1 and half are less than 1. Each wave power value represents the wave power at a specific marsh location, which is usually estimated as an average of multiple modeled wave predictions at that location using different wind directions and speeds derived from meteorological records in the area. The wind direction defines the fetch at that specific location, while the meterological record defines the frequency and intensity of the winds blowing from that direction. By combining models for the different wind directions and wind speeds at one specific location a frequency weighted average wave prediction can be made for each specific site. The wave power value therefore represents the expected average wave conditions at a specific site, given the general meteorological conditions in the area. Different sites in the same area share the same general meteorological conditions and thus, different P* values reflect different local geomorphological conditions at each site in that specific area (and not different wind directions or wind speeds).

The P* values of > 1 are sites with higher than average wave energy in the area, whereas sites with values of  < 1 have lower than average wave energy. These values provide information on the site location, in which P* values > 1 indicate more open water conditions (e.g., larger fetch, more exposure to multiple wind directions, more exposure to dominant wind direction, and/or more exposure to wind directions with stronger winds etc.) while the P* values < 1 indicate more protected locations. Thus, the relation between P* and E* shows that more protected areas are less prone to wind-generated wave erosion than more open water areas, which have more exposure to the elements. Therefore, this is best interpreted as a correlation between local geomorphological setting and erosion, and it should not be interpreted as causation between wind speed and erosion, since the entire wind speed and wind direction population was used in establishing wave power estimates; all sites in the area share the same weather and the local conditions define the wave energy response to that weather.

**Figure 1:**
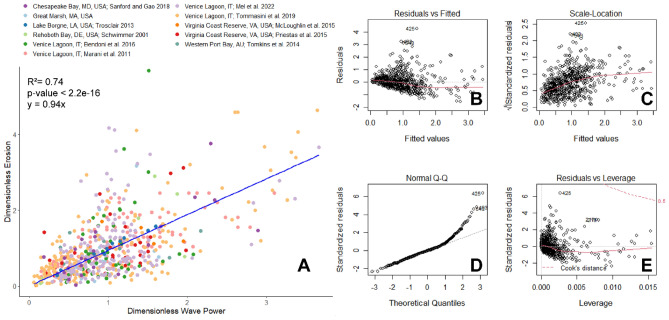
(**A**) Relationship between untransformed dimensionless wave power and dimensionless retreat. The blue line indicates the linear regression (R^2^ = 0.74, *p*-value < 2.2e-16) on the untransformed data, with an intercept of (0,0). Plots (**B**) through (**E**) show common model diagnostic plots to assess the appropriateness of the model.

When fitting a linear relationship on untransformed, dimensionless global salt marsh wave power and retreat data in the same manner as Leonardi et al.^6^, in which the intercept is forced through (0,0), the model initially shows a strong linear relationship between the two parameters (R^2^ = 0.74, *p*-value < 2.2e-16, y = 0.94x) (Fig. 1A). However, inspection of the model diagnostic plots shows heteroskedacity in the residuals versus fitted values (Fig. 1B) and the square root of standardized residuals versus fitted values (Fig. 1C), as well as non-normally distributed residuals (Fig. 1D, shown as the departure of the datapoints from the straight, dotted 1:1 line). Additionally, when performing the regression without forcing the intercept through the origin but rather allowing the intercept to be determined by the regression, the model results in a weak linear relationship (R^2^ = 0.40, *p*-value < 2.2e-16, y = 0.80x + 0.20). This unforced R^2^ (0.40) is a better measure of the correlation, and it is worth noting that forced correlations can have a very high R^2^ even if the two variables are completely independent. Linear regression models assume that the data and residuals are normally distributed, and that the residuals are independently and identically distributed with no correlation between them. The presence of heteroskedacity and non-normality in the residuals (Fig. 1B–E), as well as the fact that the original dimensionless wave power and erosion data are not normally distributed (SI Fig. 1), indicate that this linear model does not adequately nor accurately describe the relationship between the data.

**Figure 2:**
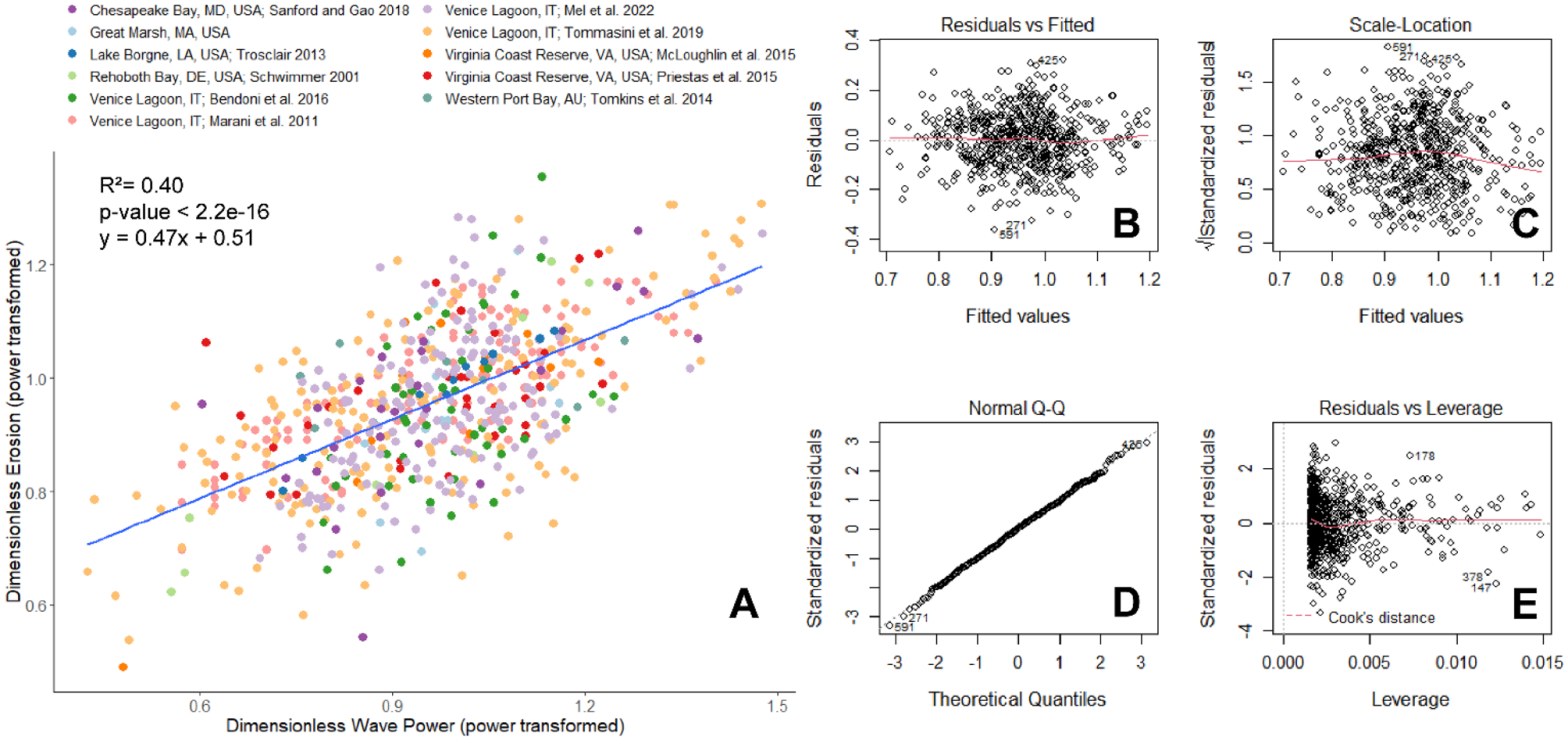
(**A**) Transformed relationship between dimensionless wave power and dimensionless retreat. The blue line indicates the linear regression (R^2^ = 0.40, *p*-value < 2.2e-16) on the power-transformed data. Plots (**B**) through (**E**) show the common model diagnostic plots.

To produce normally distributed data and residuals, a power transform was performed on the dimensionless wave power and erosion data. The linear regression on the transformed data shows a weaker, but more accurate relationship (R^2^ = 0.40, *p*-value < 2.2e-16, y = 9.47x + 0.51, where y is dimensionless erosion power-transformed by a factor of 0.175, and x is dimensionless wave power power-transformed by a factor of 0.3; Fig. 2A). When inverse-transformed and plotted on the original data and axes, the model shows a gentle power relationship between wave power and erosion (y = (0.47x^0.3^ + 0.51)^5.71^; SI Fig. 2). The diagnostic plots (Fig. 2B–E) show that there are no trends in the residuals and that they are normally distributed, which confirms it is a more robust model.

While the global relationship between wave power and erosion is a weak power relationship, when examining each salt marsh site separately, the relationship varies. For example, Table 1 shows that in Venice Lagoon the most appropriate model describing the relationship is a power curve, whereas in one study of the Chesapeake Bay^8^ the data follow an exponential curve relationship (see also SI Fig. 3). Additionally, in some sites, such as Western Port Bay and in certain sites from the Virginia Coast Reserve, wave power does not correlate well with erosion (R^2^ = 0.10, *p*-value = 0.45 for Western Port Bay, and R^2^ = 0.29, *p*-value = 0.07 for Virginia Coast Reserve).

**Table 1:** Summary of relationships between wave power and erosion described in existing salt marsh literature. The table describes whether the data are normally distributed, and if not, which transformation is most appropriate. The resulting best-fitting, most appropriate model is compared to the model described in the literature.

Data Source	Site	Transformation used on wave power	Transformation used on erosion	Best-fitting relationship	Relationship described in source literature
Marani et al. (2011)^12^	Venice Lagoon, IT	Power transform	Power Transform	Power curve (R^2^ = 0.64, *p*-value < 2.2e-16)	Linear (R^2^ = 0.55, *p*-value < 2.2e-16)
Tommasini et al. (2019)^17^	Venice Lagoon, IT	Power transform	Power Transform	Power curve (R^2^ = 0.43, *p*-value < 2.2e-16)	Linear (R^2^ = 0.52, *p*-value < 2.2e-16)
Bendoni et al. (2016)^16^	Venice Lagoon, IT	Log transform	Log Transform	Power curve (R^2^ = 0.24, *p*-value = 0.0006)	Linear (R^2^ = 0.14, *p*-value = 0.01)
Mel et al. (2022)^18^	Venice Lagoon, IT	Log transform	Log Transform	Power curve (R^2^ = 0.27, *p*-value < 2.38e-12)	Linear (When forcing regression through (0,0) as done in the original study, R^2^ = 0.66, *p*-value = 2.6e-38; when not forcing the regression, R^2^ = 0.20, *p*-value = 1.43e-8)
Priestas et al. (2015)^8^	Virginia Coast Reserve, Virginia, USA	Original data are normally distributed	Log Transform	Exponential curve (R^2^ = 0.30, *p*-value = 0.0015)	Linear (R^2^ = 0.25, *p*-value = 0.004)
McLoughlin et al. (2015)^14^	Virginia Coast Reserve, Virginia, USA	Original data are normally distributed	Original data are normally distributed	Linear	Linear (R^2^ = 0.29, *p*-value = 0.07)
Schwimmer (2001)^10^	Rehoboth Bay, Delaware, USA	Original data are normally distributed	Log Transform	Exponential curve (R^2^ = 0.80, *p*-value = 0.0012)	Power curve (R^2^ = 0.82, *p*-value = 0.0008)
Sanford and Gao (2018)^13^	Chesapeake Bay, Maryland, USA	Log transform	Log Transform	Power curve (R^2^ = 0.30, *p*-value = 0.0044)	Linear (R^2^ = 0.57, *p*-value = 1.36e-5)
Tomkins et al. (2014)^43^	Western Port Bay, AU	Original data are normally distributed	Original data are normally distributed	Linear	Linear (R^2^ = 0.10, *p*-value = 0.45)
Trosclair (2013)^44^	Lake Borgne, Louisiana, USA	Original data are normally distributed	Original data are normally distributed	Linear	Linear (R^2^ = 0.98, *p*-value = 3.60e-8)

Thus, there is no clear universal relationship, linear or otherwise, between wave power and erosion. This means no generalized conclusions can be made on when erosion mainly happens, such as during more frequent, moderate events rather than stronger storm events. Additionally, in several salt marsh sites, erosion does not correlate well to wave power, suggesting that other factors affect marsh loss^19,20^.

### Additional data from the Great Marsh, Massachusetts

In addition to a global analysis of marsh erosion, twelve sites in the Great Marsh, Massachusetts (USA), were studied from 2015 to 2020 to determine other factors that affect erosion and the relationship to wave power. In the Great Marsh, wave power (in W/m) is represented by the frequency-weighted mean of wave power over a stationary SWAN (Simulating Waves Nearshore) run, similar to the approach used by Schwimmer^10^ (see Methods for details).

**Figure 3:**
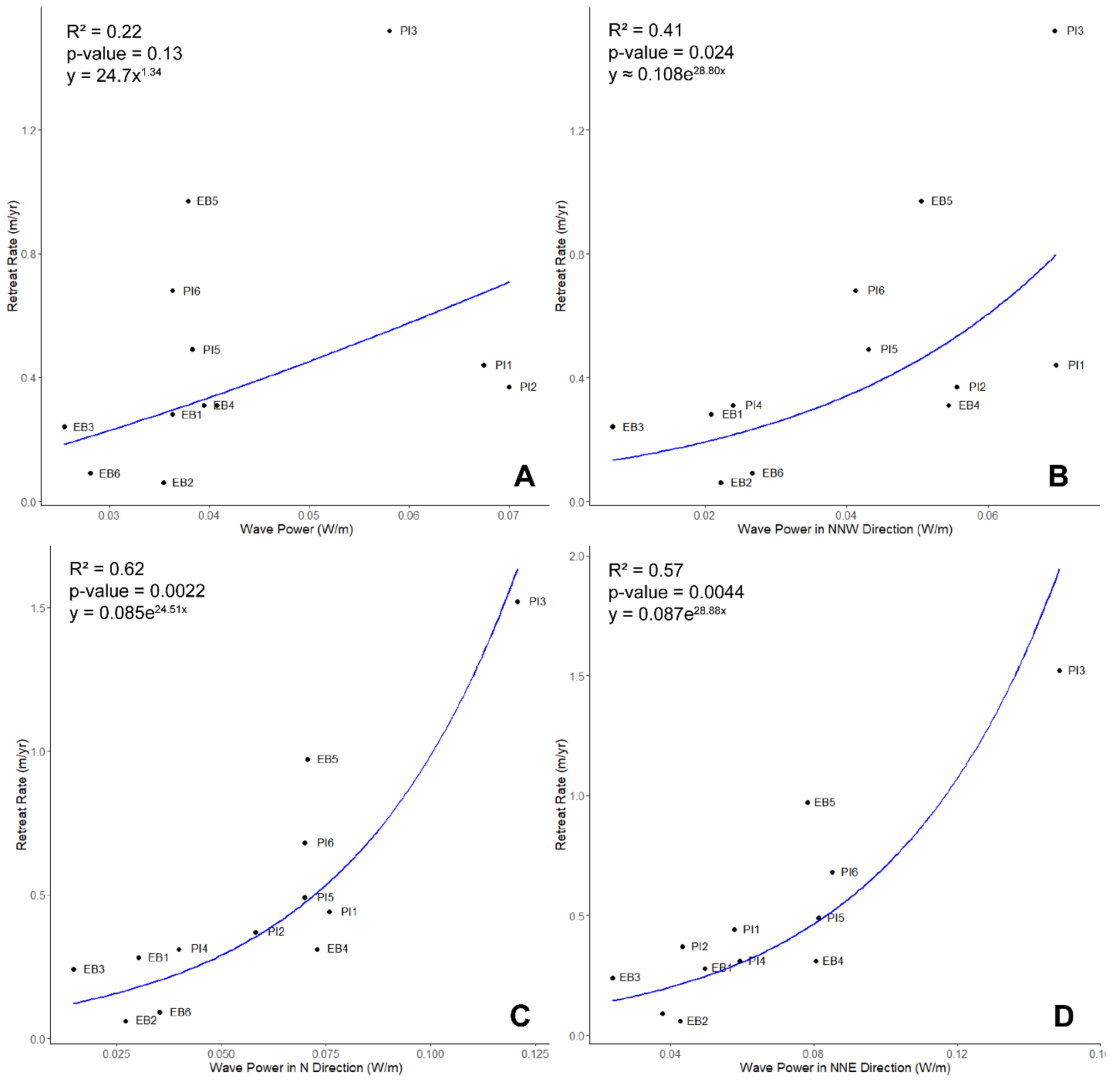
(**A**) Relationship between wave power (frequency-weighted mean from waves coming from all measured wind directions) and retreat rates. The blue line indicates the inverse-transformed linear regression (R^2^ = 0.22, *p*-value = 0.13) performed on the transformed data, resulting in a power curve. (**B**) Relationship between wave power (from frequency-weighted mean from only waves due to NNW winds) and retreat rates. The blue line indicates the inverse-transformed linear regression (R^2^ = 0.41, *p*-value = 0.024) performed on the transformed data, resulting in an exponential curve. (**C**) Relationship between wave power (from frequency-weighted mean from only waves due to N winds) and retreat rates. The blue line indicates the inverse-transformed linear regression (R^2^ = 0.62, *p*-value = 0.0022) performed on the transformed data, resulting in an exponential curve. (**D**) Relationship between wave power (from frequency-weighted mean from only waves due to NNE winds) and retreat rates. The blue line indicates the inverse-transformed linear regression (R^2^ = 0.57, *p*-value = 0.0044) performed on the transformed data, resulting in an exponential curve. Datapoint labels indicate the site in the Great Marsh.

At these Great Marsh sites there is a power curve relationship between retreat rates and the frequency-weighted mean of wave power resulting from waves coming from all wind directions, but it is not significant (R^2^ = 0.22, *p*-value = 0.13, y = 24.7x^1.34^; Fig. 3A). Relationships between erosion and wave power for each of the 16 wind directions were also analysed, and only three wind directions produced significant relationships: NNW (R^2^ = 0.41, *p*-value = 0.02, y = 0.108e^28.80x^), N (R^2^ = 0.62, *p*-value = 0.0022, y = 0.085e^24.51x^), and NNE (R^2^ = 0.57, *p*-value = 0.0044, y = 0.087e^28.88x^; Fig. 3B). The strongest winds (> 20 m/s) in this system most frequently (28% of the time) come from the NNE, followed by winds coming from the NE (20% of the time) (SI Fig. 4). Winds coming from the NNE are also overall more intense than other high energy wind directions (SI Fig. 5).

Thus, we observe that waves generated from northerly winds show improved correlation with observed erosion rates. These are relatively low frequency but higher intensity events, blowing along the axis of the bay, resulting in relatively large fetch. On the other hand, the relatively more frequent but less intense winds coming from a westerly direction, show no correlation with the erosion rate. These are winds blowing perpendicular to the axis of the bay, resulting in relatively small fetch. The winds coming from a southerly direction are more frequent than the northerly winds and share the same relatively large fetch, but these winds are relatively less intense. This wind direction does not show improved correlation with the observed erosion rate. This suggests that a combination of wind speed and direction (and thus a larger or smaller fetch) are more important than the overall frequency of a wind direction on the edge erosion rate. These findings suggest that perhaps more frequent, low to moderate energy events are not the strongest driver of marsh deterioration in this system, but rather that the marsh is vulnerable to the less frequent and higher energy events coinciding with large fetch such as Nor’easters. This conclusion, however, is specific to the Great Marsh and highlights the site-specificity of the relationships among wind/wave climate, marsh shoreline orientation, and erosion; a global linear relationship cannot accurately depict these relationships.

**Figure 4:**
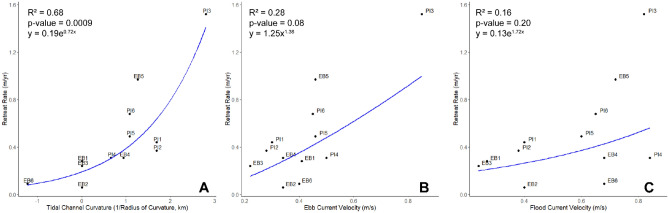
(**A**) Relationship between tidal channel curvature and retreat rates. The blue line indicates the inverse-transformed linear regression (R^2^ = 0.68, *p*-value = 0.0009) performed on the transformed data, resulting in an exponential curve. (**B**) Relationship between ebb current velocity and retreat rates. The blue line indicates the inverse-transformed linear regression (R^2^ = 0.28, *p*-value = 0.08) performed on the transformed data, resulting in a power curve. (**C**) Relationship between flood current velocity and retreat rates. The blue line indicates the inverse-transformed linear regression (R^2^ = 0.16, *p*-value = 0.20) performed on the transformed data, resulting in an exponential curve. Datapoint labels indicate the site in the Great Marsh.

Whereas there is a significant nonlinear relationship between marsh retreat and wave power from the NNE, N, and NNW directions in the Great Marsh, other parameters can also help explain or are correlated to marsh retreat. Channel curvature has been used to link channel flow and the morphology and migration of fluvial channels^21^. It has been shown to translate to tidal settings as well^22^. Several of the Great Marsh sites are located at the thalweg of the main channels with relatively narrow mudflats for shelter, allowing for tidal currents to directly impact the marsh edge. Tidal channel curvature has a strong, significant relationship with retreat rates in the Great Marsh (R^2^ = 0.68, *p*-value = 0.0009, y = 0.19e^0.72x^; Fig. 4A). This relationship suggests tidal channels may also play a significant role in marsh retreat in this mesotidal system. However, modeled ebb and flood current velocities do not reflect that same strong relationship (R^2^ = 0.28, *p*-value = 0.08, y = 1.25x^1.38^ for ebb current velocities, Fig. 4B; and R^2^ = 0.16, *p*-value = 0.20, y = 0.13e^0.72×^ for flood current velocities, Fig. 4C). A multiple linear regression including ebb and flow velocities, as well as channel curvature, produces only a marginally better fit (R^2^ = 0.72, *p*-value = 0.01) and the ebb and flow velocity parameters are not significant (*p*-values < 0.05). As a result, the influence of tidal channels on marsh edge retreat may not be directly due to flow velocities, but perhaps other factors such as the imbalance in radial pressure along a channel bend and centrifugal force acting on the bank^23^. Current-driven erosion clearly plays a role in the Great Marsh system in addition to wind-driven edge erosion, and tidal influences should be considered in future analyses and models of marsh edge erosion, particularly for mesotidal systems such as this one.

Since marshes are heterogeneous, specific sites can have an outsized impact on the relationships. While site PI3 looks like a statistical outlier, no outliers were removed from this analysis because each site represents real processes occurring on the marsh, rather than an error in data collection. In fact, site PI3 exemplifies how rates of erosion are not constant across the marsh due to heterogeneous processes. Moreover, when the site was removed it did not improve the models but rather worsened them in some cases, resulting in erroneous models showing retreat and wave power to be linearly related. This highlights the importance of including data that represent a range of marsh processes and settings, from protected areas with low retreat rates to highly exposed areas with very high retreat. This variability resulting from heterogeneity of marsh properties and processes shows that generalized relationships are often limited and may not appropriately advance understanding and prediction of marsh retreat. Global, generalized relationships should not be used to predict the fate of marsh edge erosion, but rather local models that are calibrated using comprehensive, site-specific field data.

### Factors influencing the wave power-marsh retreat relationship

When determining relationships among marsh retreat and potential processes that influence retreat, it is important to treat the data in a statistically appropriate manner before performing analyses to ensure no statistical assumptions are violated. Here, we show that while there is indeed a significant relationship between wave power and marsh retreat at the global level, and at the individual marsh level in several cases, the relationship is not always linear (Fig. 2 and Table 1). Although a linear relationship between erosion and wave power suggests marsh resilience to extreme weather events that produce large waves and exponentially higher energy conditions^6^, marsh-specific studies demonstrate a more complex relationship.

In fact, storms have been found to better correlate with marsh retreat than all-weather or fair-weather conditions. For example, in the Greater Thames area (UK), erosion was found to be related to changes in the wind/wave climate and more extreme water levels and storm waves, as well as localized human activities^24^. In Lake Borgne, a nonlinear response of erosion was observed during a single extreme event, the passage of Hurricane Isaac^25^. In this marsh, erosion occurred mostly before the peak of the simulated hurricane and before the marsh was fully submerged. This is consistent with findings from Tonelli et al., who suggested wave thrust on the marsh edge depends on tidal level, with thrust increasing with rising water level and subsequently decreasing once the marsh is submerged^7^. In the Great Marsh, wave power coming from major storm directions (NNE and N), in addition to wave power coming from the more moderate NNW direction, correlated with retreat while a weighted average of wave power for all directions did not (Fig. 3), indicating that storms actually do have an impact on retreat in this system, with the impact growing exponentially with wave power. Thus, in this setting, there is no resilience to frequent, strong storm events as suggested by the previous generalized linear relationship^6^.

In many marshes, the relationship between wave power and retreat is weak or not significant. While bank failure and the resulting bank retreat is associated with wave forcing^26^, mass failure events can also add noise to the data and weaken the relationship; slumping does not fully correlate with instantaneous wave power, because mass failure can occur in calm conditions, including through soil creep^27^. Likewise, bank instability that results in slumping can be caused or enhanced by hydrodynamic forcing due to erosive forces overcoming an intrinsic erosion threshold^16^. While hydrodynamic forces impact the marsh edge instantaneously, the resulting morphological evolution may only be observed at longer (i.e., decadal) timescales, showing a time-delayed linkage between processes acting on the edge and the morphological form that the edge takes^28^. For example, in the Great Marsh, site EB2 eroded only after a major storm, which resulted in a very low long-term retreat rate that was not fully reflected in the importance of wave power impacting the site. However, the impact of mass failures on the relationship should weaken when analyzing retreat rates at the time scale of multiple years^18^. Moreover, although mass failures may obscure the relationship between hydrodynamic forcing and retreat, they are an important process that can account for most of the retreat characterizing some marshes^8^.

Salt marshes can exhibit considerable spatial heterogeneity in intrinsic factors such as vegetation characteristics and geotechnical properties, at both the scale of an individual site and the entire marsh^17,29–31^. Thus, this heterogeneity can weaken the relationship between external forcings such as wave power and marsh retreat. For example, the variability in this relationship from site to site has been attributed to crab bioturbation and local marsh resistance to erosion^8^, clamming and other localized human activities^24^, local variations in edge morphology that impact the local exposure to waves^14^, varying bulk density and bank height^12^, varying adjacent water depths^17^, and the presence of vegetation versus bare marsh edge^5,32^. In addition to heterogeneity of intrinsic factors, spatial differences in hydrodynamics such as wind-driven fluctuations in water levels can cause spatial asymmetry in erosion^33^. The geometry of the marsh margin has also been found to relate to spatial differences in retreat rates, in which marsh edge type and configuration affects the hydrodynamic characteristics impacting the edge, which in turn impacts the morphological evolution of the edge^28^. In the Great Marsh, the strong link between retreat rates and tidal channel curvature (Fig. 4A) suggests that factors other than wave forcing are significantly influencing erosion and shoreline retreat, such as potentially tidal forcing or spatial variability in edge morphology or intrinsic factors.

Due to the heterogeneity of salt marshes, even with multiple levels of normalization of the data, there is no universal relationship linking retreat and wave power. In some marsh systems, the relationship may be significant, but it can vary from a linear to power or exponential relationship (Table 1). Additionally, in some marshes, there is no significant relationship whatsoever between wave power and retreat; processes other than wave forcing may be responsible for marsh areal loss, such as low sediment accretion rates relative to RSLR^34^.

Thus, it is important to consider the complexities of each marsh system when measuring or modeling marsh retreat. Unlike previously suggested models, there is no single wave power-retreat relationship that can be applied to predict or explain marsh loss, and the response or vulnerability of marshes to storms or other factors is often marsh-specific and nonlinear. The heterogeneity of marshes and processes affecting them means that generalized or universal relationships are often not useful and can lead to misinterpretations and false predictions of marsh loss. The actual, valid relationships between wave power and erosion are often weaker and nonlinear, and highly site-specific. When modeling marsh loss, it is therefore necessary to train and test any relationship on local data to confirm the unique, marsh-specific relationship among retreat and potential processes causing erosion. Finally, we also emphasize the importance of ensuring the appropriateness and robustness of regressions performed when determining these relationships.

## Methods

### Great Marsh physical setting

**Figure 5:**
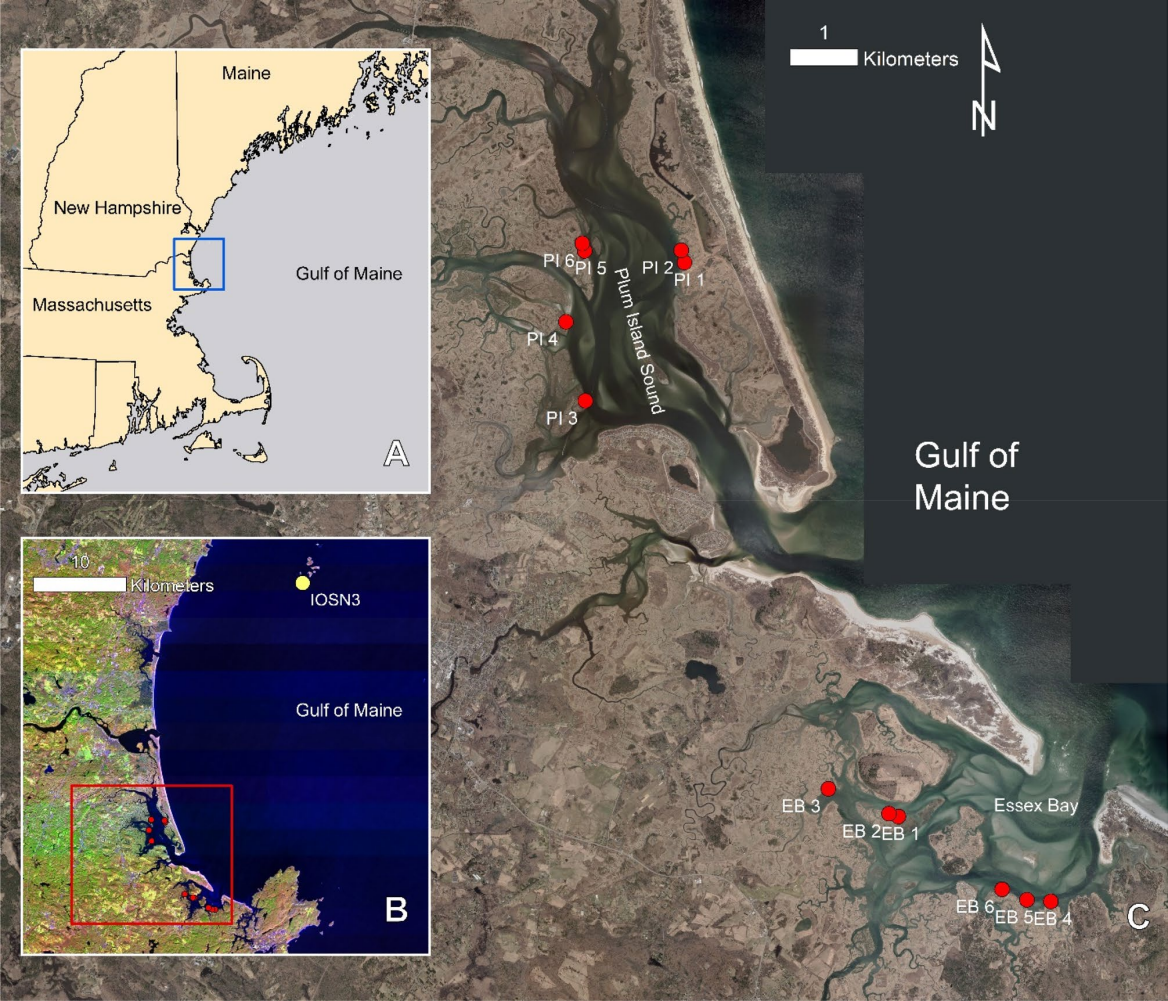
Map of Great Marsh study site. (**A**) Map of larger region; blue box shows map extent of (**B**) map indicating location of NOAA buoy IOSN3 (yellow dot) and study sites (red dots); red box indicates map extent of (**C**) Great Marsh, Massachusetts; red dots show locations of the study sites. Satellite imagery for panel B is from USGS Landsat 9, available under public domain. Imagery for panel C is from MassGIS 2019 Aerial Imagery, available under public domain. Map was made using Esri ArcGIS Desktop 10.8.2.

The Great Marsh is an expansive marsh system in northern Massachusetts, USA, comprising several small estuaries in Plum Island Sound and Essex Bay (Fig. 5). This back-barrier marsh has a tidal range of 2.6 to 2.8 m and consists of marsh and large open water areas with sandy shoals. The estuaries in the system provide low suspended sediment input^35^, and the mean grain size across the marsh is approximately 24% sand, 58% silt, and 17% clay^36^.

**Figure 6:**
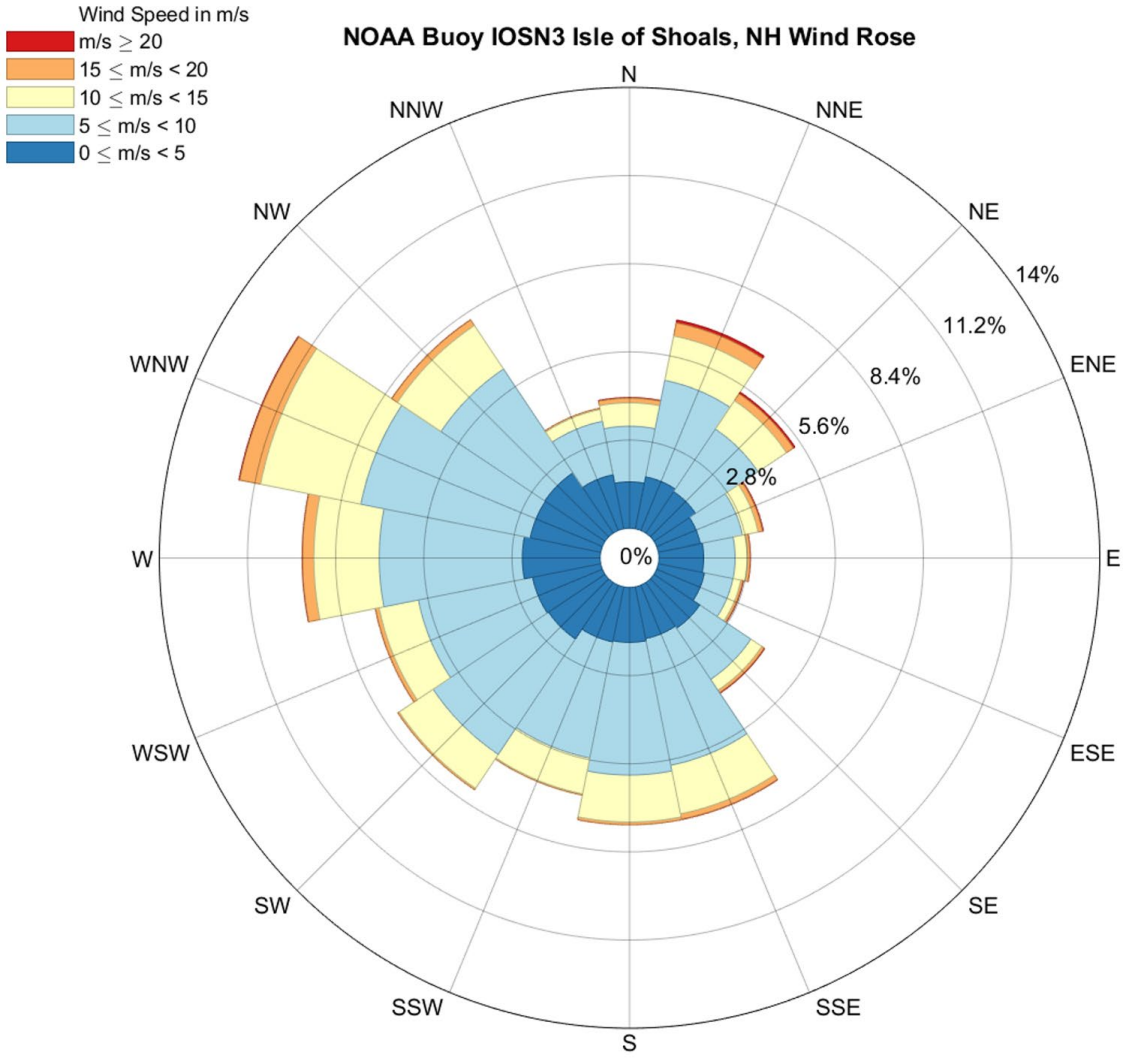
Wind rose of wind data used, from NOAA Buoy IOSN3 located in Isle of Shoals, New Hampshire.

The marsh platform is dominated by high marsh species *Spartina patens* and *Distichlis spicata*, with smaller areas of low marsh and tidal creek edges dominated by *Spartina alterniflora*. The relative sea level trend in this region is 2.9 mm/yr (from NOAA Boston Harbor tidal gauge 8,443,970, based on 1921–2021 record). Prevailing winds in this region come from the WNW and W, and strong gales and storm winds (> 20 m/s) come primarily from the NNE and NE (Fig. 6).

### Great Marsh retreat rates

Twelve marsh edge sites were surveyed repeatedly over time using Real-Time Kinematic GPS (RTK-GPS) (SI Table 1). Sites were chosen to represent the diversity of marsh edge found, from sections exposed to the bay or sound or sheltered behind islands, to sites eroding predominantly either through mass failure or through continuous, particle by particle erosion. At each site, the marsh edge was surveyed using the RTK-GPS covering approximately 70 m of shoreline. Each site was surveyed at least three times, in the summer of 2015, in 2016, and either 2019 or 2020. The RTK-GPS points were processed and corrected using the NOAA Online Positioning User Service (OPUS), and retreat rates were calculated using the linear regression rate from the Digital Shoreline Analysis System (DSAS), a software add-in for Esri ArcGIS desktop which calculates rate-of-change statistics from multiple historic shoreline positions^37^. A mean of 53 transects were used at each site to calculate the retreat rates. DSAS retreat rates were also validated with field retreat data; three evenly spaced rebars were placed on the marsh platform at each site, and the distance from the marsh edge to the rebar was recorded over the same timeframe as the RTK surveys.

### Great Marsh wave characteristics

Significant wave heights impacting the Great Marsh study sites were estimated using Simulating Waves Nearshore (SWAN). SWAN is a numerical wave model that provides estimates of wave parameters in coastal and estuarine areas from given bottom and wind conditions^38,39^. For this study’s model, wind conditions were derived from the NOAA data buoy station IOSN3 in Isle of Shoals, New Hampshire, based on the 1996–2020 data record. A 20 m × 20 m bathymetric grid for the study region was created by combining NOAA hydrographic survey data (from NOAA NCEI Bathymetric Data Viewer), 2013–2014 USGS lidar data^40^, and extensive single-beam sonar data collected in the field. To ensure all the sites of interest were exposed to waves in the simulation, the datum of the bathymetric grid was set to MHHW, or 1.47 m above MSL in this region. SWAN was run in stationary mode for 16 different wind directions (shown in the wind rose; Fig. 6) and for 4 different wind speed bins: 5–10 m/s, 10–15 m/s, 15–20 m/s, and winds faster than 20 m/s, resulting in 64 simulations.

Wave energy (in J/m^2^) was calculated from the significant wave heights computed by SWAN using the following formula,

$$E = \frac{1}{16} \rho gH_{sig}^{2} ,$$

Where *E* is wave energy, *ρ* is the density of water, *g* is the acceleration due to gravity, and *H*_*sig*_ is the significant wave height. Wave power (in W/m), also called wave energy flux^14^ or wave power density^12^, was calculated using the following formula,

$$P_{w} = Ec_{g} ,$$

Where *P*_*w*_ is the wave power or energy flux, and *c*_*g*_ is the group velocity, calculated through the expression,

$$c_{g} = { }\frac{c}{2}\left[ {1 + { }\frac{2kD}{{{\text{sinh}}\left( {2kD} \right)}}} \right]$$

Where *c* is the celerity, *k* is the angular wave number, and* D* is the water depth.

Weighted means of wave height, energy, and power were calculated at each site using the frequency of wind speed and direction conditions determined using NOAA buoy data (Fig. 6).

### Great Marsh tidal channel and current characteristics

In fluvial settings, radius of curvature is used to study meanders and migration. Radius of curvature is the reciprocal of curvature and is measured as the radius of an arc that best fits the curve. Knighton explained the relationship between fluvial migration/erosion rates and radius of channel curvature normalized by channel width, showing a link between channel flow, morphology, and migration^20^. Finotello et al. found that observed Venice Lagoon channel migration rates of tidal meanders were similar to fluvial meanders, suggesting that tidal meanders in some marsh systems may not be as stable as conventionally viewed, and that similar measures such as radius of curvature can be applied to tidal channels^22^. In the Great Marsh study sites, three sites had a straight tidal channel, and thus the radius of curvature was infinite. As a result, for this study channel curvature was used instead of radius of curvature; a straight channel thus had a channel curvature of 0, and channels that were concave at the study site had a positive curvature value while channels that were convex had a negative curvature value. Channel curvature was measured by approximating the channel curves based on satellite imagery using circle arcs and measuring the arc’s radius, and taking the reciprocal.

Tidal current velocities were extracted from hydrodynamic models previously developed and calibrated for tidal harmonics using field observations^41,42^. Using the calibration simulations, which covered a typical 30-day simulation, peak flood, and ebb velocities were extracted during spring tide conditions. Velocities were extracted at the nearest “wet” model grid cell adjacent to the sites where retreat rates were measured, to eliminate wet-dry perturbations from influencing tidal currents. Peak ebb and peak flood velocities were then averaged over the three largest tidal excursions during spring tide conditions and were subsequently used in correlations with marsh retreat data.

### Global retreat rates and wave power

Existing literature providing wave power values and retreat rates for salt marshes globally were surveyed^3,8,10,12,14,18–20,43,44^, and the data were synthesized to determine the overall relationship between wave power and erosion. Following the approach outlined in^6^, to remove the between-site and between-study variability of these values, the data were normalized with the following formula:

$$P^{*} = \frac{P}{{P_{avg} }}{\text{ and }}E^{*} = { }\frac{E}{{E_{avg} }}$$

Where P* and E* are the normalized, dimensionless wave power and erosion values, respectively; P and E are the individual wave power and erosion values, respectively; and P_avg_ and E_avg_ are the site-specific, mean wave power and erosion values for each study, respectively.

### Statistics

While high R^2^ values are often used to show that a model is “good,” linear models may be invalid if they do not meet the assumptions of a linear regression, i.e. normality of data and residuals, and no heteroskedacity or correlations in the residuals. A robust, valid model meets these assumptions. Normality of data was assessed using the Shapiro–Wilk test (normal data have a *p*-value > 0.05), as well as visual inspection of histograms and normal Quantile–Quantile plots of the sample quantiles. If the data were not normal, they were transformed to create normal distributions. Linear regressions were then performed on the normally distributed data, and the models were assessed by inspection of residuals versus fitted values plots, normal quantile–quantile plots of the standardized residuals, scale-location plots, and residuals versus leverage plots. Robust models showed no trend in the residuals (i.e., no heteroskedacity or clear linear correlation) and showed a normal distribution (the normal Quantile–Quantile plot was a straight line). Presence of heteroskedacity in the residuals was determined using the Breusch-Pagan test, in which a *p*-value < 0.05 indicates heteroskedacity. If the data had to be transformed, the resulting linear model for the data was inverse-transformed to fit the original data; for example, if the data required a power transform to perform a linear regression, the model was inverse-transformed to a resulting power relationship that fit the original data.

## Supplementary Information

Supplementary Information.

## Data Availability

The data generated during this study are included in this published article in the supplementary information and are also available at the NOAA National Centers for Environmental Information (NCEI) archive (NCEI Accession Number 0210237). All other data analysed was from previously published articles, referenced in this paper, and are available via the original publications.
