# Supplementary material for: Reevaluating the wave power-salt marsh retreat relationship
Source: Sci Rep. 2023 Feb 18;13:2884. doi: 10.1038/s41598-023-30042-y (PMC9938853; doi:10.1038/s41598-023-30042-y)
Supplement: Supplementary file 1 — Supplementary Information. [file 41598_2023_30042_MOESM1_ESM.pdf]

Reevaluating the wave power-salt marsh retreat relationship **- Supplementary Information**

Houttuijn Bloemendaal, L.J.[1*], FitzGerald, D.M.[1], Hughes, Z.J.[1], Novak, A.B.[1], Georgiou, I.Y.[2]
[1]Boston University Department of Earth and Environment; [2]The Water Institute of the Gulf

*Corresponding author

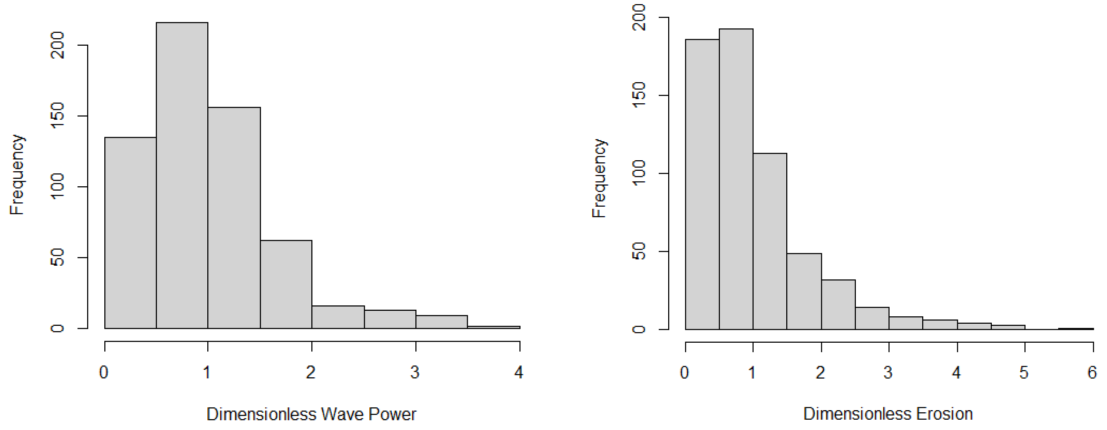

SI Fig. 1 Distributions of global dimensionless wave power and erosion data.

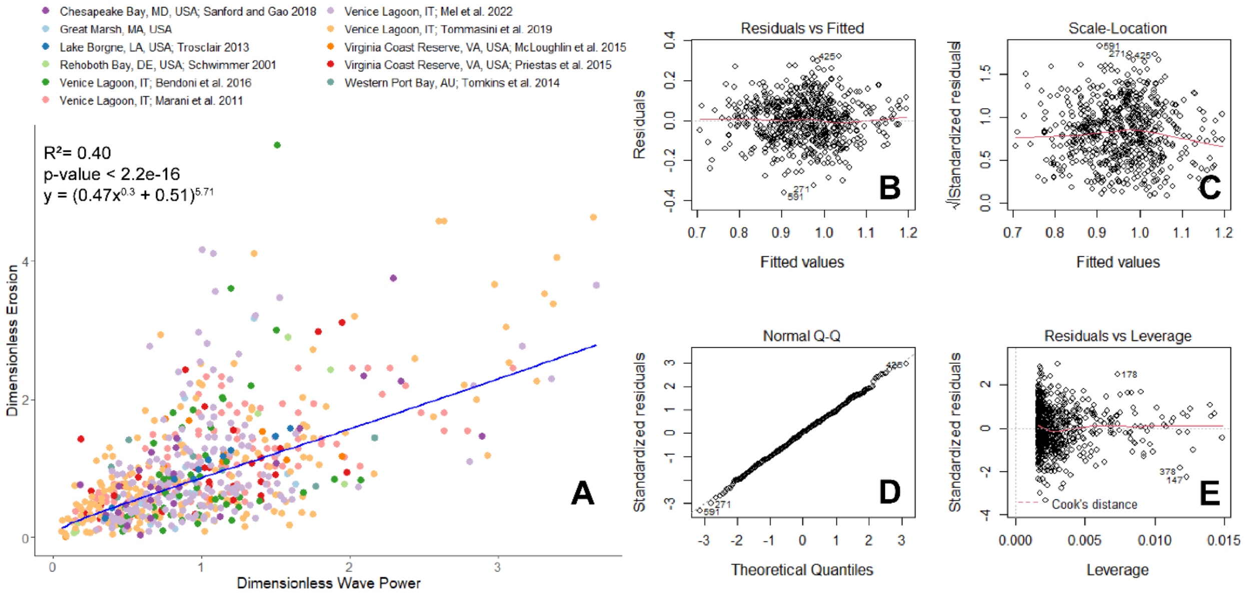

SI Fig. 2 A. Linear regression on the power-transformed dimensionless wave power and erosion data; the linear regression is subsequently inverse-transformed to produce a power curve and is plotted on the original, untransformed data. Plots B through E show common model diagnostic plots to assess the appropriateness of the model.

Marani et al. (2011)

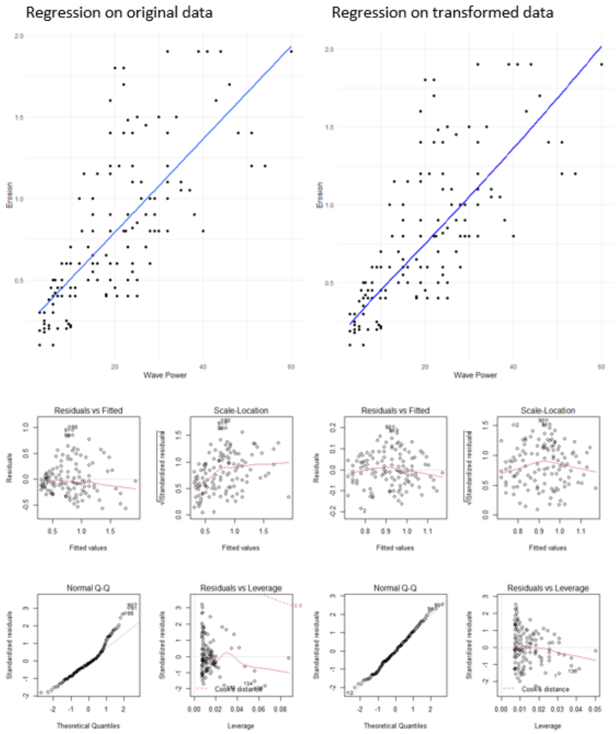

Tommasini et al. (2019)

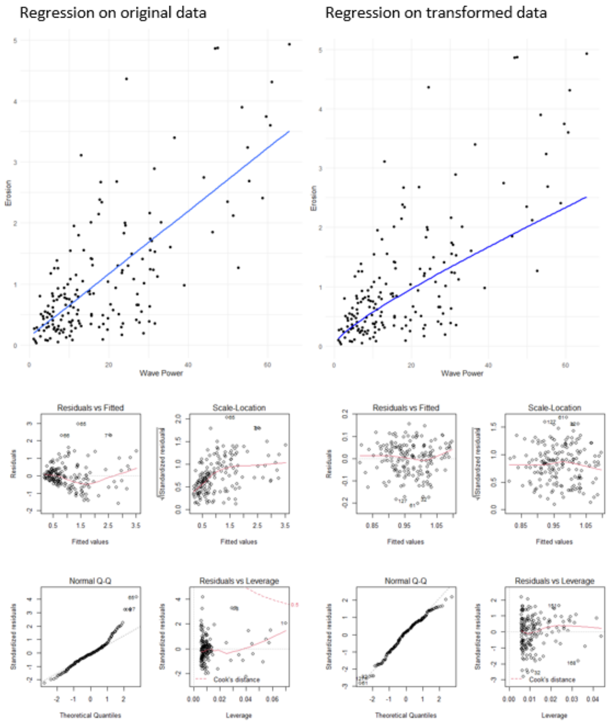

Bendoni et al. (2016)

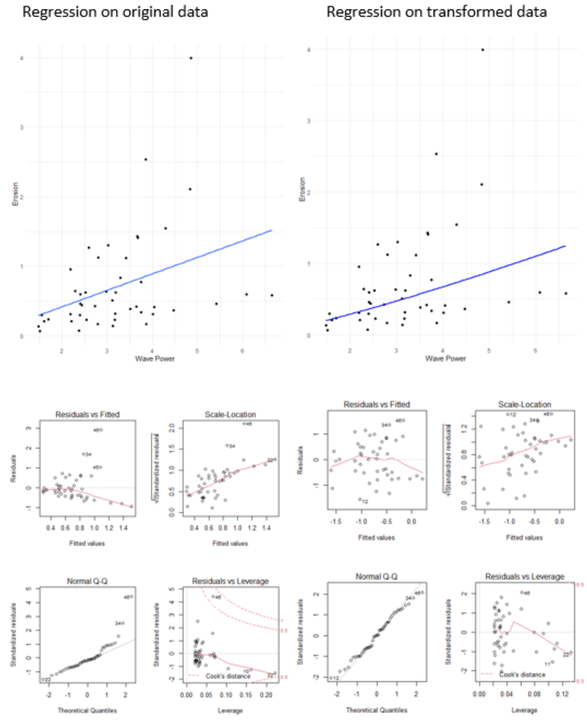

Mel et al. (2022)

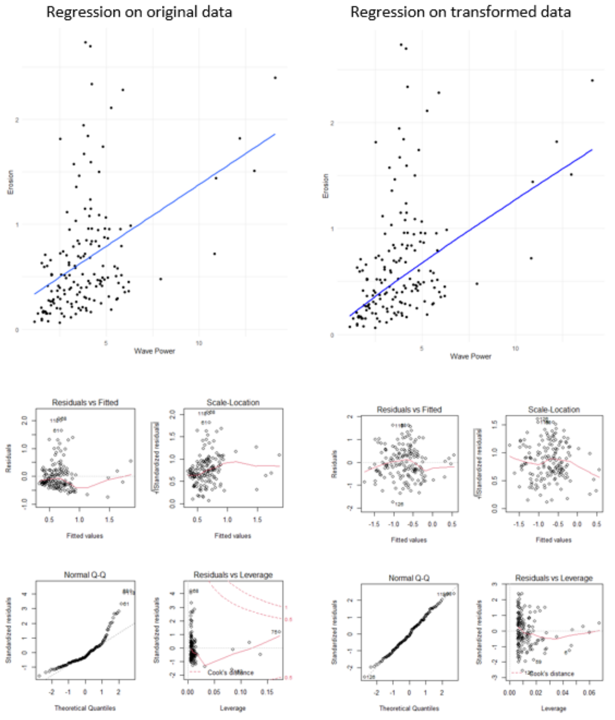

Priestas et al. (2015)

Regression on original data

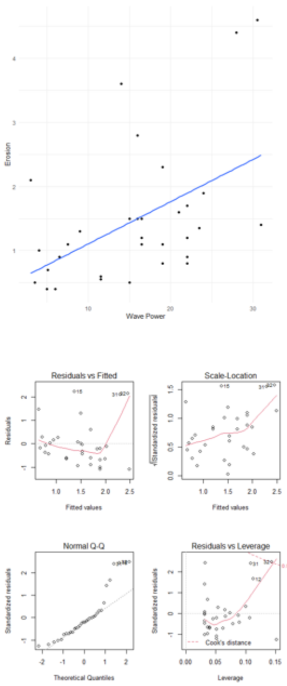

Regression on transformed data

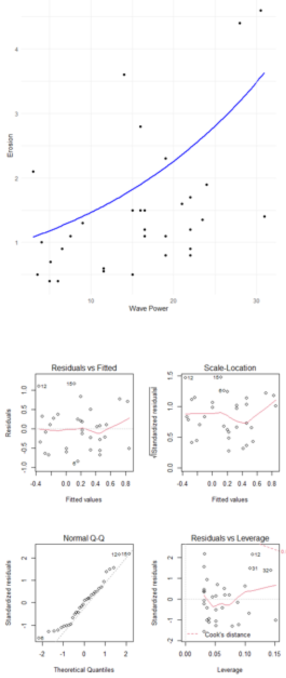

Sanford and Gao (2018)

Regression on original data

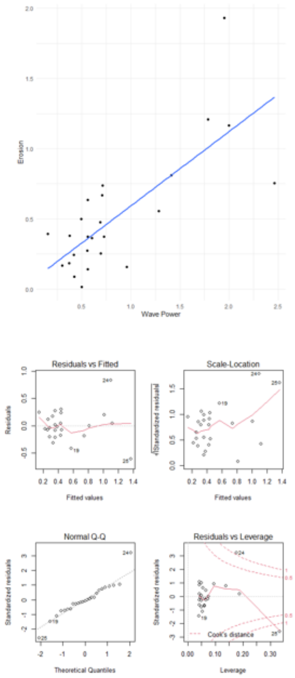

Regression on transformed data

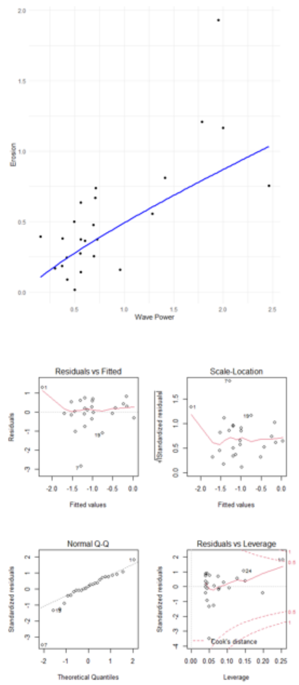

Schwimmer (2001)

Regression on original transformed data (semi-log transform)

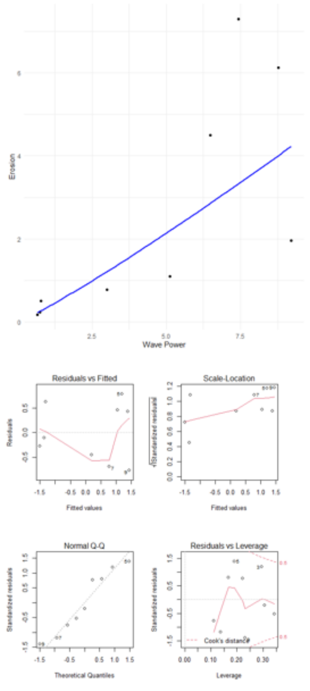

Regression on newly transformed data (log-log transform)

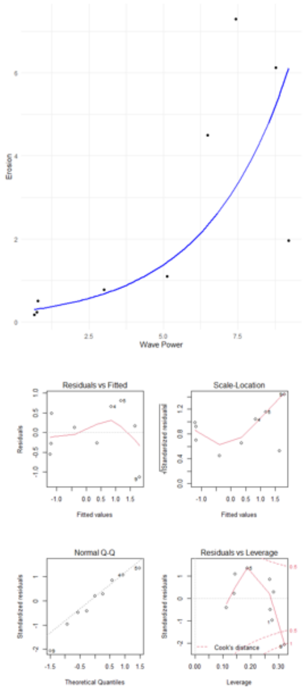

Regression on untransformed data

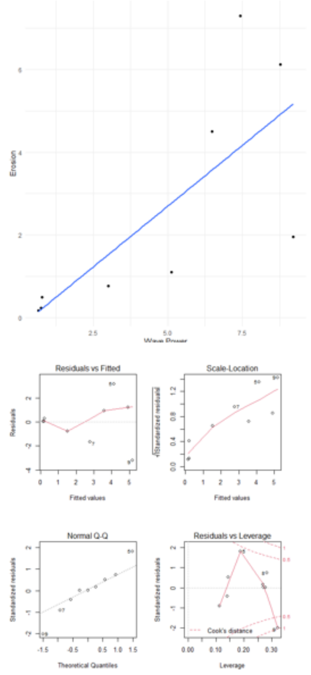

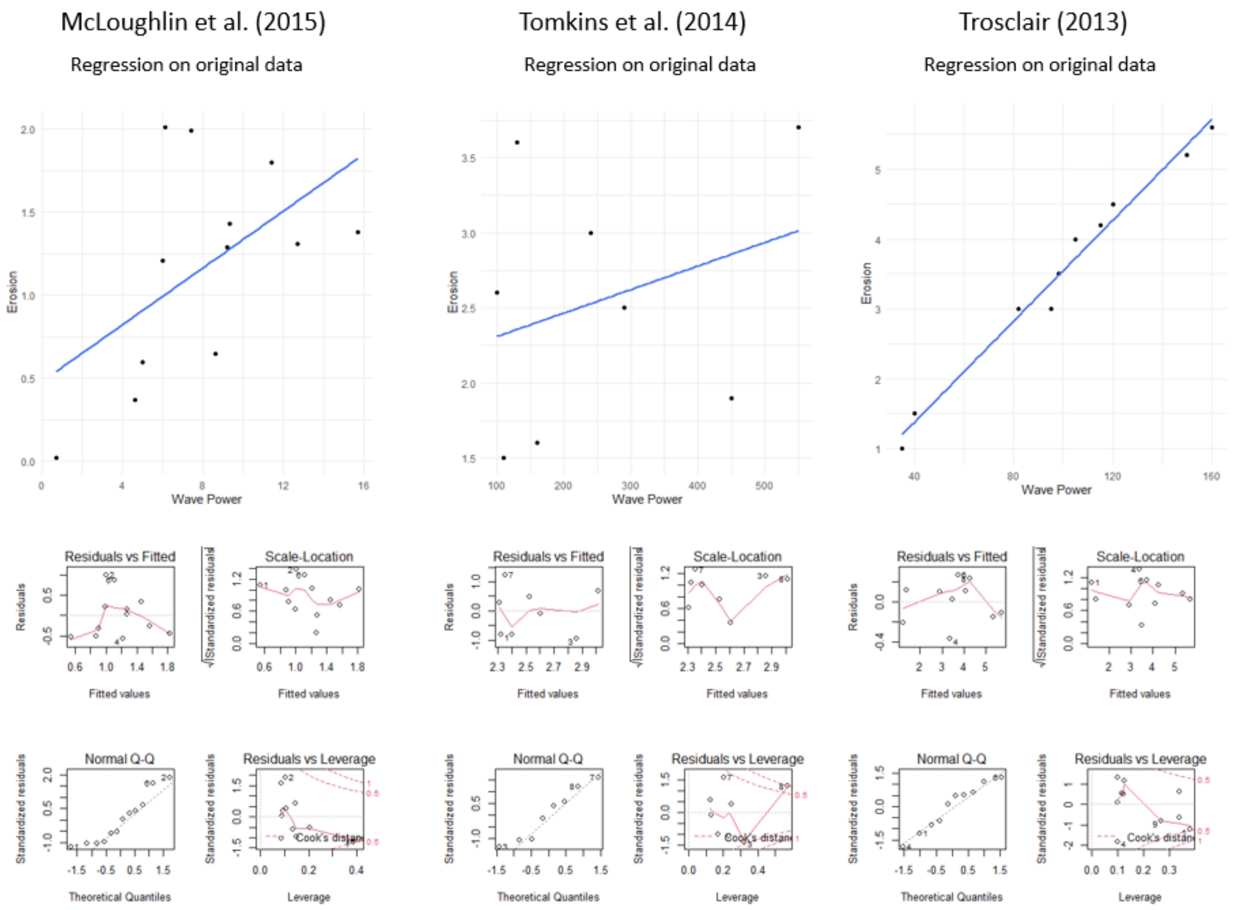

SI Fig. 3 Comparison of models from surveyed literature and models on the transformed data. Y axis on scatter plots is erosion, x axis is wave power.

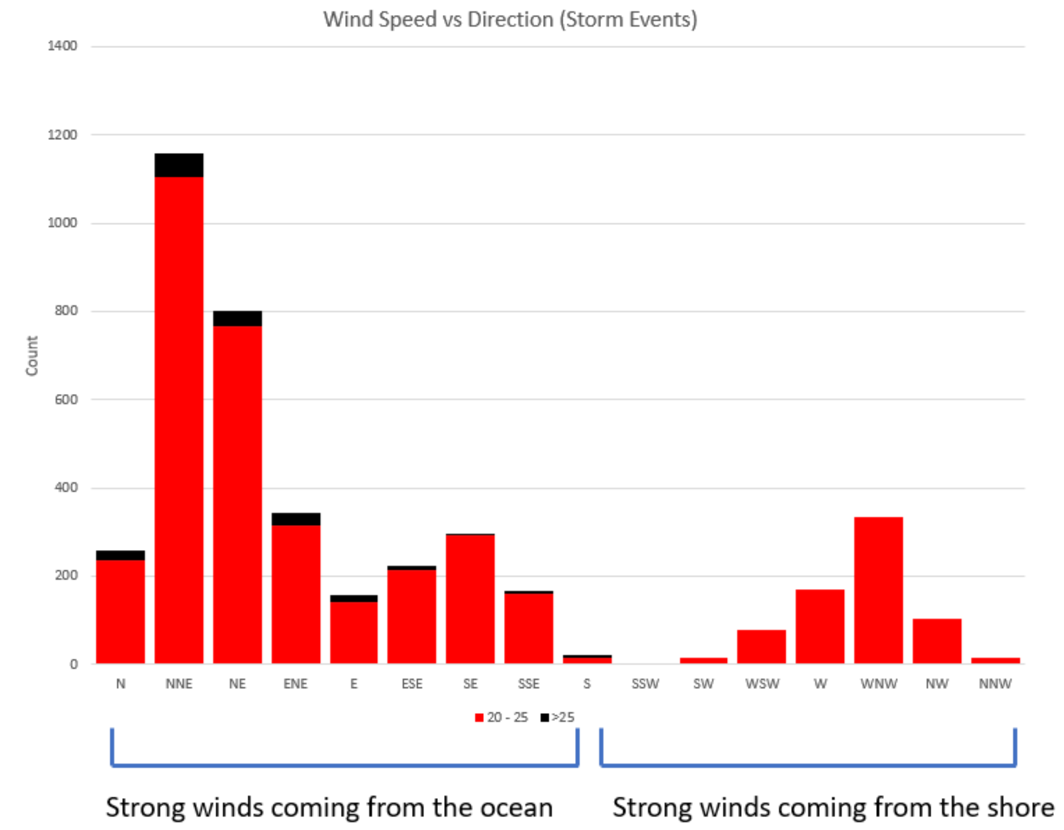

SI Fig. 4 Bar chart of number of high speed wind events, separated by direction wind is blowing from. Red indicates wind speeds between 20 and 25 m/s, and black indicates wind speeds of greater than 25 m/s. Strong winds most frequently come from the NNE.

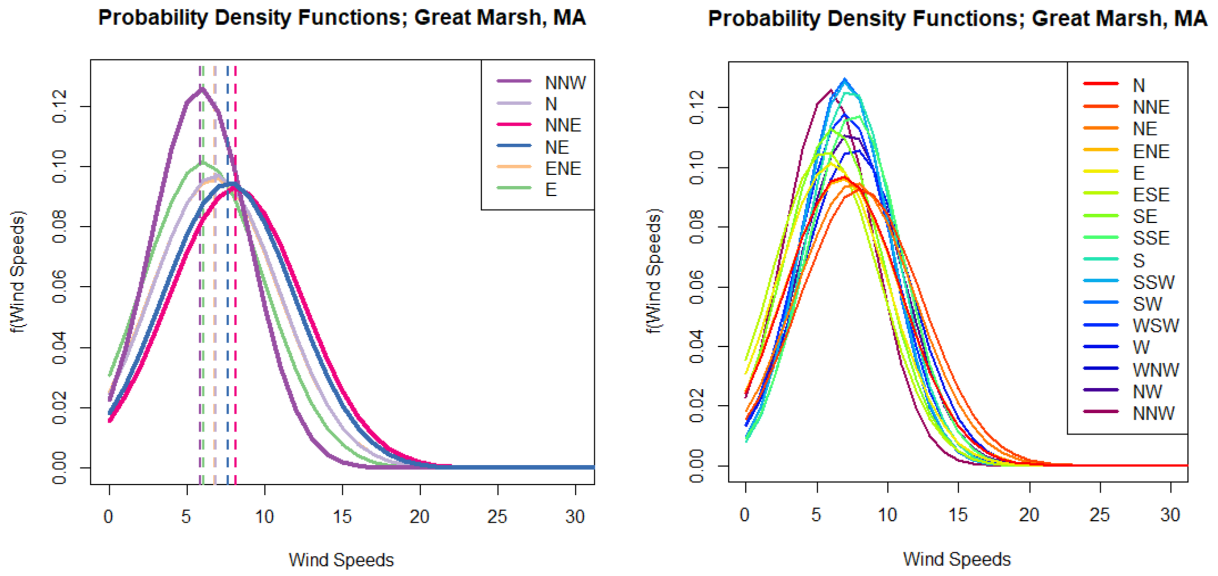

SI Fig. 5 Left: Probability density functions for wind directions with the most frequent strong winds, as well as the more frequent and moderate NNW direction as the relationship between wave power and retreat in this wind direction was found to be significant (along with the N and NNE directions). The dashed lines represent the mean of each distribution. Right: Probability density functions for all wind directions.

Site	Retreat Rate (m/yr)	Uncertainty (m/yr)	Number of Transects Used	Length of Shoreline Surveyed (m)	Dates of Shoreline Surveys
**EB1**	0.28	0.39	54	69	08/25/2015, 03/18/2016, 11/20/2019
**EB2**	0.06	0.3	61	80	08/25/2015, 03/18/2016, 11/21/2019
**EB3**	0.24	0.08	44	66	08/25/2015, 03/18/2016, 11/20/2019
**EB4**	0.31	0.07	59	87	08/25/2015, 01/15/2016, 11/25/2019
**EB5**	0.97	0.39	29	65	08/25/2015, 01/15/2016, 10/08/2019, 10/08/2020
**EB6**	0.09	0.19	38	76	08/25/2015, 01/15/2016, 10/08/2019
**PI1**	0.44	0.13	61	76	08/26/2015, 03/31/2016, 05/10/2016, 07/27/2020
**PI2**	0.37	0.09	75	98	08/26/2015, 03/31/2016, 05/10/2016, 07/27/2020
**PI3**	1.52	0.42	46	62	08/26/2015, 04/28/2016, 07/23/2020
**PI4**	0.31	0.15	52	58	08/26/2015, 04/28/2016, 07/27/2020
**PI5**	0.49	0.07	62	67	08/26/2015, 05/10/2016, 07/23/2020
**PI6**	0.68	0.3	49	56	08/26/2015, 05/10/2016, 07/23/2020

SI Table 1. Summary of shoreline retreat surveys used.

Site	Wave Power (All Wind Directions; W/m)	Wave Power (N Wind Direction; W/m)	Wave Power (NNE Wind Direction; W/m)	Wave Power (NE Wind Direction; W/m)	Wave Power (ENE Wind Direction; W/m)	Wave Power (E Wind Direction; W/m)	Wave Power (ESE Wind Direction; W/m)	Wave Power (SE Wind Direction; W/m)	Wave Power (SSE Wind Direction; W/m)	Wave Power (S Wind Direction; W/m)
**EB1**	0.036	0.030	0.050	0.058	0.069	0.065	0.053	0.035	0.034	0.032
**EB2**	0.035	0.027	0.043	0.054	0.071	0.071	0.060	0.037	0.031	0.027
**EB3**	0.026	0.015	0.024	0.031	0.049	0.062	0.062	0.047	0.047	0.037
**EB4**	0.04	0.073	0.081	0.065	0.045	0.030	0.024	0.015	0.013	0.016
**EB5**	0.038	0.071	0.078	0.070	0.058	0.042	0.032	0.023	0.022	0.019
**EB6**	0.028	0.035	0.038	0.036	0.034	0.029	0.024	0.020	0.020	0.021
**PI1**	0.068	0.076	0.058	0.019	0.011	0.008	0.009	0.018	0.037	0.054
**PI2**	0.07	0.058	0.043	0.015	0.011	0.009	0.014	0.029	0.057	0.077
**PI3**	0.058	0.121	0.149	0.122	0.117	0.109	0.102	0.071	0.047	0.023
**PI4**	0.041	0.040	0.059	0.062	0.070	0.073	0.073	0.063	0.059	0.043
**PI5**	0.038	0.070	0.081	0.069	0.063	0.063	0.067	0.061	0.062	0.042
**PI6**	0.036	0.070	0.085	0.071	0.066	0.064	0.063	0.054	0.052	0.035

Site (Continued)	Wave Power (SSW Wind Direction; W/m)	Wave Power (SW Wind Direction; W/m)	Wave Power (WSW Wind Direction; W/m)	Wave Power (W Wind Direction; W/m)	Wave Power (WNW Wind Direction; W/m)	Wave Power (NW Wind Direction; W/m)	Wave Power (NNW Wind Direction; W/m)	Flood Current Velocity (m/s)	Ebb Current Velocity (m/s)	Channel Curvature (1/Radius of Curvature; km)
**EB1**	0.029	0.026	0.027	0.033	0.039	0.030	0.021	0.27	0.41	0.00
**EB2**	0.025	0.023	0.026	0.034	0.041	0.032	0.022	0.40	0.34	0.00
**EB3**	0.028	0.021	0.020	0.014	0.013	0.009	0.007	0.24	0.22	0.00
**EB4**	0.017	0.019	0.026	0.039	0.055	0.056	0.054	0.68	0.34	0.94
**EB5**	0.018	0.016	0.019	0.029	0.045	0.048	0.050	0.72	0.46	1.26
**EB6**	0.021	0.016	0.023	0.031	0.038	0.032	0.027	0.68	0.40	-1.23
**PI1**	0.066	0.071	0.076	0.093	0.115	0.097	0.070	0.40	0.30	1.68
**PI2**	0.088	0.088	0.085	0.092	0.104	0.082	0.056	0.38	0.28	1.68
**PI3**	0.020	0.020	0.023	0.030	0.045	0.057	0.069	0.82	0.85	2.80
**PI4**	0.034	0.028	0.024	0.027	0.032	0.027	0.024	0.84	0.50	0.65
**PI5**	0.024	0.010	0.003	0.006	0.025	0.038	0.043	0.60	0.46	1.08
**PI6**	0.021	0.010	0.004	0.005	0.023	0.035	0.041	0.65	0.45	1.08

SI Table 2. Great Marsh, MA hydrodynamic and related data.
